# Vaccination Against SARS-CoV-2 in Lung Transplant Recipients: Immunogenicity, Efficacy and Safety

**DOI:** 10.3389/fimmu.2022.906225

**Published:** 2022-06-01

**Authors:** Monika Svorcova, Rene Novysedlak, Robert Lischke, Jiri Vachtenheim, Zuzana Strizova

**Affiliations:** ^1^Third Department of Surgery, Prague Lung Transplant Program, First Faculty of Medicine, Charles University and University Hospital Motol, Prague, Czech Republic; ^2^Department of Immunology, Second Faculty of Medicine, Charles University and University Hospital Motol, Prague, Czech Republic

**Keywords:** COVID-19, mRNA vaccination, Pfizer, Moderna, transplant, immunosuppression

## Abstract

Lung transplant (LuTx) recipients are considered to be at higher risk of developing serious illness from COVID-19. COVID-19 vaccines were shown in randomized clinical trials to substantially reduce the severity of COVID-19, however, patients receiving immunosuppressants were excluded from these trials. Observational studies report a proportion of solid organ transplant (SOT) recipients being able to mount sufficient titers of SARS-CoV-2 specific IgG antibodies, however, other studies demonstrate that more than 90% of the SOT recipients elicit neither humoral nor cellular immune response after vaccination. Currently, the third booster dose of the COVID-19 vaccines was shown to elicit strong immune responses and may, thus, represent a potent tool in the prevention of severe COVID-19 infection in SOT recipients, including patients after lung transplantation. To address the main challenges of SARS-CoV-2 vaccination in LuTx recipients in the era of COVID-19, we have closely collected all available data on the immunogenicity, efficacy and safety of COVID-19 vaccines in LuTx recipients.

## Introduction

Immunosuppressive therapy prevents both acute and chronic rejection in a significant proportion of solid organ transplant (SOT) recipients and to date, calcineurin inhibitors, cell cycle inhibitors, and corticosteroids belong to the most commonly-prescribed anti-rejection drugs for the treatment of lung transplant (LuTx) patients (1). Yet, bacterial, viral, and fungal infections make up the largest proportion of serious adverse events associated with anti-rejection therapy (2). For that reason, lung transplant (LuTx) recipients are considered to be at higher risk of developing serious illness from COVID-19 (3).

The field of organ transplantation has been severely affected in the era of COVID-19 (4). Most of the SOT centers have experienced a significant decline in transplant activity given a variety of factors, such as limited donor pools, extensive SARS-CoV-2 testing, and decreased intensive care unit (ICU) capacities (4, 5). Lung transplantations were associated with a number of donor-derived COVID-19 infections which raised the urge to set novel guidelines for the prevention and treatment of LuTx recipients (4, 5).

COVID-19 vaccines in SOT recipients could provide at least a certain level of protection and in randomized clinical trials, COVID-19 vaccines were shown to substantially reduce the severity of COVID-19 (6, 7). Vaccination against COVID-19 was strongly encouraged but initially, patients receiving immunosuppressants were excluded from the randomized clinical trials (8, 9). For that reason, the development of protective immunity after COVID-19 vaccination in SOT recipients has been investigated mostly throughout observational and/or real-life evidence studies (10). While several studies evaluated the serological responses in patients receiving post-transplant medication, little is still known about the mechanisms of protective immunity and the duration of such protection (11–14). The interpretation of the vaccination efficacy based on serological responses bears certain limitations, such as highly variable antibody titers and kinetics among individuals (15, 16). The strict cutoff values that are being applied by most antibody tests may not efficiently classify the seropositivity as negative control values may overlap true positive low-titer values (17). Furthermore, it is crucial to investigate virus-neutralizing antibody responses in vaccinated individuals, including those after lung transplantation. Virus neutralizing antibodies serve as a reliable marker of protection against COVID-19 and were previously well-established in other viral infections (18, 19). Previous experiences with influenza vaccination in LuTx recipients reported pre-transplant patients being able to mount a more vigorous immune response as compared to LuTx recipients (20). Similarly, COVID-19 vaccination is currently recommended primarily in waitlisted patients to achieve a stronger humoral and cellular immune response (21). Even-thought the re-exposure may sustain protective antibodies, it is yet to be clarified which factors have affect the adaptive immune responses (15).

The fear of suboptimal immune response is often accompanied by the fear of severe adverse events in SOT recipients (22). Although the benefit of COVID-19 vaccination outweighs the risk of adverse events, several acute rejections have been reported in SOT recipients which raises a number of safety concerns (23–25). On the other hand, most of the COVID-19 studies in SOT recipients show no association between the vaccination and the development of organ rejection (22).

In this review, we attempted to address the main challenges of SARS-CoV-2 vaccination in LuTx recipients in the era of COVID-19. We have closely collected all available data on the immunogenicity, efficacy and safety of COVID-19 vaccines in LuTx recipients to provide a deeper insight into this complex and challenging issue.

## Methods

We conducted a comprehensive review of the literature on the immunogenicity, efficacy and safety of COVID-19 vaccines in LuTx recipients. COVID-19, lung transplantation, SARS-CoV-2, vaccine, and vaccination, were used as the keywords in the search strategy. The following databases were used: Medline/PubMed, Scopus, and Web of Science. Only English-written and peer-reviewed studies that were published in indexed international journals were reviewed.

## Anti-COVID-19 Vaccination in Lung Transplant Recipients: First and Second Doses

Randomized clinical trials have evaluated the immunogenicity of diverse anti-SARS-CoV-2 vaccines, however, the efficacy of anti-SARS-CoV-2 vaccines in immunosuppressed patients, including those receiving anti-rejection drugs, remains to be clarified (10).

It has been already demonstrated that patients that were vaccinated while on the transplant waiting list were able to mount much higher anti-SARS-CoV-2 immune responses as compared to patients that were vaccinated after the transplantation (5, 21).

In a study by Narasimhan et al., evaluating serological responses to two doses of COVID-19 mRNA vaccine in LuTx recipients, only 25% of LuTx recipients mounted SARS-CoV-2 specific IgG response with 36% of responders in the Moderna cohort and 19% of responders in the Pfizer cohort (11). Also, the antibody titers in the LuTx recipients were significantly lower as compared to non-transplanted COVID-19-naïve subjects (11). These data suggested that in the majority of LuTx patients, receiving two doses of the anti-COVID-19 mRNA vaccine did not guarantee protection against a COVID-19 infection. Furthermore, the Moderna vaccine was found to generate a trend towards a more robust humoral response than the Pfizer vaccine in LuTx patients (7).

A prospective observational cohort study by Shostak et al. evaluated the immunogenicity and safety of the BNT162b2 vaccine in a total of 168 LuTx recipients (8). This was one of the first studies to investigate the generation of the humoral immune response after COVID-19 vaccination in LuTx recipients. Interestingly, the SARS-CoV-2 specific IgG antibodies were detected in only 4% of the study participants after the first dose, and in 18% of the study participants after the second dose of the mRNA vaccine. The participants who were receiving either antimetabolites or mammalian target of rapamycin (mTOR) inhibitors were less likely to develop sufficient antibody titers. These findings were in accordance with a study by Boyarsky et al. demonstrating diminished humoral responses in SOT recipients receiving antimetabolites (26). Despite the fact that neither the virus-neutralizing antibodies nor the cellular immune response was evaluated in the study by Shostak et al., the safety of the BNT162b2 vaccine in LuTx recipients was carefully assessed and only minor adverse events, such as injection site reactions, were observed.

Another study addressing both the humoral and cellular immune response to the BNT162b2 vaccine in 50 transplant patients (42 heart Tx, 7 LuTx, and one heart-lung Tx) was conducted by Schramm et al. (27) In this study, 96% of the study participants displayed non-detectable anti-SARS-CoV-2 antibodies 21 days after the first dose. Moreover, 90% of the SOT recipients elicited neither humoral nor cellular immune response three weeks after the two doses of the BNT162b2 vaccine (27). Interestingly, in a small subgroup of transplant recipients where humoral response was non-detectable, a slight IFN-γ release was observed. Although these findings could indicate a possible T-cell mediated protection in SOT recipients after COVID-19 vaccination, the authors also state a possibility of cross-reactivity with a former coronavirus infection.

In a study by Hallet et al., vaccine-elicited immune responses of 103 LuTx recipients and 134 heart transplant recipients were evaluated (12). In this study, all tested subjects had dramatically decreased humoral responses as compared to the general population. Of 103 LuTx recipients, 9% responded to the first dose, 27% responded to the second dose and 64% were classified as non-responders. Heart transplant recipients were more likely to mount detectable humoral responses (12). In this study, SOT recipients receiving anti–metabolite maintenance immunosuppression therapy were less likely to mount protective antibody titers after vaccination and these diminished antibody responses were in dire contrast with clinical trials in the general population. Severe adverse events, such as acute rejection or anaphylaxis, were not reported throughout the study (8).

The immunogenicity of the BNT162b2 mRNA COVID-19 vaccine was also evaluated in 48 LuTx recipients where none of the study participants had detectable anti-SARS-CoV-2 specific IgG antibodies after the first, nor after the second vaccine dose (16). Interestingly, these results were in contrast with the LuTx recipients infected with COVID-19 where humoral responses were observed in 85% of the patients within 90 days following the COVID-19 diagnosis (28). However, only four out of twelve patients exhibited SARS-CoV-2 specific CD4^+^ and CD8^+^ T cells (28). The analysis of the safety outcomes has not been assessed in this study, however, one patient did not receive a second vaccine dose due to acute rejection.

The authors Yanis et al. correlated the humoral and cellular immune responses after two doses of COVID-19 mRNA vaccine in 56 SOT patients and 26 healthy individuals (29). In this study, four LuTx recipients were included, however, the majority of SOT types were kidney (41%) and liver (37%). The authors presented only 21.6% of the SOT patients eliciting comparable antibody responses to healthy individuals.

A single-center retrospective study by Bolinelli et al. was designed to evaluate the clinical effectiveness of COVID-19 vaccines in LuTx patients by assessing the respiratory symptoms, oxygen saturations, imaging findings and other clinical variables, in SARS-CoV-2 positive LuTx patients with/without COVID-19 vaccination (30). The study has shown lower levels of inflammatory markers during the acute phase of COVID-19 and lower risk of COVID-19 complications, such as respiratory failure or ventilation need, in vaccinated individuals.

## Anti-COVID-19 Vaccination in Lung Transplant Recipients: Third Dose

Since the two doses of COVID-19 vaccine did not elicit strong immune responses in SOT recipients, the administration of the third vaccine dose was initiated. Subsequently, several observational studies reported the immunogenicity and safety of the third dose in LuTx recipients (28).

In a study by Kamar et al., 101 consecutive SOT recipients were given a third dose of the BNT162b2 mRNA vaccine. In this study, eight LuTx recipients were included and the administration of a third dose significantly promoted the increase in antibody titers and was proven as a reliable booster of the vaccine immunogenicity (31). Patients with only limited humoral response were mostly older and had a higher degree of immunosuppression. The authors, however, highlight that although the vaccine immunogenicity was significantly improved with the third dose, the clinical efficacy in SOT recipients is yet to be clarified. Therefore, SOT recipients should still avoid COVID-19 exposure and encourage their relatives to receive an anti-COVID-19 vaccination (19).

The authors Hall et al. conducted a randomized clinical trial in 120 SOT recipients to evaluate the immunogenicity of the third dose of the vaccine compared to a placebo. In this study, the third-dose booster substantially improved the immunogenicity of the mRNA-1273 vaccine (32). In addition, the median percent virus neutralization was 71% in patients receiving the Moderna vaccine as compared to 13% in patients receiving the placebo. The third dose administration was safe and well-tolerated in all SOT study participants. The authors reported neither cases of acute organ rejection nor serious adverse events in the SOT recipients within the study period (20).

Since there are currently only limited data on the efficacy, immunogenicity and safety of the third dose of anti-COVID-19 vaccine in LuTx recipients, promising results demonstrated by Peled et al. may further promote attempts to evaluate these outcomes in individuals after lung transplantation (21). In this study, the humoral response increased from 23% to 67% after the third dose of the BNT162b2 vaccine in heart transplant recipients (33). Moreover, the vaccination with a third dose was not associated with any severe side effects, nor with organ rejection (33).

Safety concerns regarding the third dose application were raised in a review by Ison et al. (34) The study by Ou et al. demonstrated an increased risk of organ rejection in a cohort receiving three doses of COVID-19 vaccine as compared to the two-dos cohort. However, the conclusions were based on one case of rejection per cohort (23).

## Discussion

Vaccination against COVID-19 is an essential step in the prevention of severe COVID-19 infection in LuTx recipients (28). Currently, LuTx candidates are recommended to undergo vaccination while on the transplant waiting list (5, 21). These recommendations stem from the fact that the COVID-19 vaccine immunogenicity, efficacy and safety were shown to be greater in the pre-transplant patients as compared to the SOT recipients (5, 8, 35). Similar findings were previously reported in LuTx patients receiving influenza vaccination prior transplantation (20, 36).

The immunogenicity of anti-SARS-CoV-2 vaccines in patients receiving anti-rejection drugs varies (10). Several studies report a proportion of SOT recipients being able to mount sufficient titers of SARS-CoV-2 specific IgG antibodies, while other studies show that more than 90% of the SOT recipients elicit neither humoral nor cellular immune response after vaccination (27). Furthermore, SOT recipients receiving antimetabolites were shown to have rather limited humoral responses to COVID-19 vaccination (8, 26).

LuTx recipients were less likely to develop humoral immune response as compared to healthy individuals, but also as compared to heart transplant recipients (12). However, SARS-CoV-2 reactive T cells were observed in a small number of LuTx recipients (28).

Interestingly, the third booster dose of the COVID-19 vaccines was shown so far to elicit strong immune responses and may, thus, represent a potent tool in the prevention of severe COVID-19 infection in SOT recipients (33). The safety concerns regarding COVID-19 vaccines in LuTx recipients seem to be outweighed by the benefits of vaccination, and rare cases of organ rejection in SOT recipients do not provide a rationale for withholding the vaccination (22).

The clinical efficacy of COVID-19 vaccines in LuTx recipients is yet to be elucidated. To date, large cohort studies attempted to provide real-life evidence of COVID-19 vaccines efficacy in diverse SOT recipients. These studies show almost 80% reduction of symptomatic COVID-19 illness in vaccinated individuals (37). However, in lung transplantation, more data are needed to understand the extent of immune protection.

## Author Contributions

MS, RN, JV and ZS contributed to the conceptualization, data curation, and writing of the original draft. MS, RN, RL, JV and ZS contributed to the review and editing of the final draft. All authors contributed to the article and approved the submitted version.

## Funding

This study was supported by the Ministry of Health, Czech Republic – Conceptual Development of Research Organization, University Hospital Motol, Prague, Czech Republic (No. 6028), and by the Cooperatio Program, Research Area SURG.

## Conflict of Interest

The authors declare that the research was conducted in the absence of any commercial or financial relationships that could be construed as a potential conflict of interest.

## Publisher’s Note

All claims expressed in this article are solely those of the authors and do not necessarily represent those of their affiliated organizations, or those of the publisher, the editors and the reviewers. Any product that may be evaluated in this article, or claim that may be made by its manufacturer, is not guaranteed or endorsed by the publisher.
